# Safety and efficacy of paclitaxel plus carboplatin versus paclitaxel plus cisplatin in neoadjuvant chemoradiotherapy for patients with locally advanced esophageal carcinoma: a retrospective study

**DOI:** 10.1186/s13014-022-02190-4

**Published:** 2022-12-30

**Authors:** Li Jiang, Jie Zhu, Xue Chen, Yi Wang, Lei Wu, Gang Wan, Yongtao Han, Xuefeng Leng, Lin Peng, Qifeng Wang

**Affiliations:** 1 School of Medicine, University of Electronic Science and Technology of China, Chengdu, China; 2 Department of Radiation Oncology, Radiation Oncology Key Laboratory of Sichuan Province, Sichuan Cancer Hospital & Institute, Sichuan Cancer Center, School of Medicine, University of Electronic Science and Technology of China, 55 South Renmin Ave, Fourth Section, Chengdu, 610041 Sichuan China; 3 Department of Thoracic Surgery, Sichuan Cancer Hospital & Institute, Sichuan Cancer Center, School of Medicine, University of Electronic Science and Technology of China, Chengdu, China

**Keywords:** Neoadjuvant chemoradiotherapy, Esophageal squamous cell carcinoma, Locally advanced esophageal squamous cell carcinoma, Carboplatin, Cisplatin, Paclitaxel, Adverse effects, Overall survival, Progression-free survival

## Abstract

**Background and purpose:**

We evaluated and compared the efficacy and safety of chemotherapy with paclitaxel plus cisplatin (TP) or carboplatin (TC) in patients with locally advanced esophageal squamous cell carcinoma (LA-ESCC) who underwent neoadjuvant chemoradiotherapy (NCRT).

**Materials and methods:**

This single-center retrospective study assessed patients with LA-ESCC (cT2N + M0, cT3-4aNanyM0) receiving NCRT plus curative-intent esophagectomy with TP or TC regimen. The primary endpoints were grade ≥ 3 adverse events (AEs) and overall survival (OS). AEs were compared using a t-test according to CTCAE 4.0. The Kaplan–Meier survival curves were compared using the log-rank test; the treatment effect was measured using hazard ratios and 95% confidence intervals.

**Results:**

We included 151 and 50 patients in the TC and TP groups, respectively. Baseline demographic and clinical characteristics were well balanced between groups. The TP group exhibited significantly higher hematologic and non-hematologic AEs than the TC group, and the noticeable difference was the incidence of febrile neutropenia of grade 3 or higher (*P* = 0.011). No significant intergroup differences were noted considering postoperative complications, resection margins, or pathological complete remission rate (all *P* > 0.05). OS and progression-free survival (PFS) did not significantly differ between groups. The estimated 3-year OS and PFS rates were 65.1% versus 69.4% and 58.4% versus 53.5% for TP and TC groups, respectively.

**Conclusion:**

In patients with LA-ESCC, we recommend TC, not TP, as an optimal chemotherapy regimen for NCRT, given its superiorsafety profile and comparable efficacy.

**Supplementary Information:**

The online version contains supplementary material available at 10.1186/s13014-022-02190-4.

## Introduction

Esophageal cancer is the eighth most common malignancy and the sixth leading cause of cancer-related deaths worldwide [1]. In Asian countries, including China and Japan, esophageal squamous cell carcinoma (ESCC) is the predominant histological type [2], and locally advanced disease was the most common stage in newly diagnosed patients with esophageal cancer. Neoadjuvant chemoradiotherapy (NCRT) followed by radical esophagectomy is the standard treatment for locally advanced ESCC (LA-ESCC) owing to its survival benefits [3–5].

Several studies have confirmed that the most suitable dose of NCRT for esophageal cancer is 39.6–45.0 Gy [6–8], and a fractionated dose of 40 Gy is currently recommended as the standard dose in neoadjuvant radiotherapy [9]. Following the publication of the CROSS trial, the chemotherapy regimen paclitaxel plus carboplatin (TC) was widely applied in NCRT for LA-ESCC [10]. TC regimens have gained considerable popularity owing to their potential for low toxicity. Meanwhile, a meta-analysis has indicated that NCRT with paclitaxel plus cisplatin (TP) was more effective than cisplatin plus 5-fluorouracil (PF) in LA-ESCC [11]. However, cisplatin is known to be associated with severe side effects, including ototoxicity, neurotoxicity, and myelosuppression [12, 13]. It should be noted that the TP regimen may result in poor tolerance to neoadjuvant treatment owing to associated adverse events (AEs). However, the optimal neoadjuvant chemotherapy regimen remains poorly established, given the lack of head-to-head randomized controlled trials (RCTs).

In the present single-center retrospective study, we comprehensively reviewed the safety and efficacy profiles of the TP and TC regimens in patients with LA-ESCC undergoing standard NCRT. The purpose of this study was to compare the safety of TP and TC regimens by comparing treatment-related AEs and the efficacy of TP and TC regimens, considering pathological complete remission (pCR) rate, radical resection rate (R0 resection rate), and 3-year overall survival (OS) and progression-free survival (PFS).

## Method

### Patients

Herein, we retrospectively reviewed patients with LA-ESCC who had received NCRT followed by surgery at Sichuan Cancer Hospital and Institute between May 2017 and June 2021. The inclusion criteria were as follows: 1. Newly diagnosed patients with histologically confirmed resectable LA-ESCC; LA-ESCC, defined as pathologically proven ESCC, with clinical stages of TNM classification cT1-2 N + M0 or cT3–cT4aNanyM0, according to the American Joint Committee on Cancer Staging System (UICC-AJCC) 8th edition [14]; 2. Eastern Cooperative Oncology Group (ECOG) performance score of 01 [15]; 3. Patients who received NCRT followed by resectional surgery; 4. TC and TP chemotherapy regimens used were employed. Exclusion criteria were as follows: 1. Patients who had received prior treatment for primary tumors or nodes; 2. All non-squamous cell carcinomas, including adenocarcinoma or small cell carcinoma;. Patients who received chemotherapy only, radiotherapy only, or neither before surgery; 4. Patients treated with NCRT only and did not undergo surgery; 5. Patients who received sequential chemoradiotherapy, 6. Patients whose chemotherapy regimen induced unknown changes. This retrospective analysis was approved by the appropriate institutional review board.

### Chemotherapy regimens

The TP group regimen consisted of paclitaxel 135–175 mg/m^2^ (day 1) and cisplatin 75 mg/m^2^ (day 1–3) at weeks 1 and 4 for 2 cycles, or paclitaxel 50 mg/m^2^ (day 1) and cisplatin 30 mg/m^2^ (day 1) administered weekly during radiation therapy (RT) for 4 cycles. The TC group regimen comprised a paclitaxel dose of 135–175 mg/m^2^ (day 1) and carboplatin administered at an area under the curve of 35 mg/mL/min (day 1) at weeks 1 and 4 for 2 cycles. The dose of chemotherapy regimens and dose adjustments were determined by medical oncologists.

### Radiotherapy scheme

Gross tumor volume (GTV) was contoured according to clinical imaging investigations such as esophagoscopy, computed tomography (CT), and positron emission tomography-computed tomography (PET-CT). The clinical target volume (CTV) was defined as the GTV plus a 2–3-cm margin in the cranial-caudal direction and a 0.5-cm margin in the transverse plane, without the CTV boundary exceeding anatomical barriers, such as blood vessels. The median total radiotherapy dose was 40 Gy (2.0 Gy per fraction). Intensity-modulated RT (IMRT) was concurrently initiated with the first cycle of chemotherapy and was administered 5 days per week during 4–5 weeks of radiotherapy.

### Safety evaluation

AEs and postoperative complications were used to evaluate safety. Acute AEs (occurring within 3 months after NCRT) during chemoradiation were graded according to the Common Terminology Criteria for Adverse Events version 4.0. The severity of RT-induced esophagitis and pneumonitis was graded according to the Radiation Therapy Oncology Group (RTOG) acute radiation morbidity scoring criteria [16]. Postoperative complications were defined as grade ≥ 3 AEs according to the Clavien-Dindo classification [17].

### Efficacy evaluation

Effectiveness was assessed using the pCR rate, R0 resection rate, and 3-year OS and PFS. pCR was defined as the absence of gross or microscopic tumor tissue in both the primary lesion and lymph nodes upon examining the surgical specimen. R0 was defined as microscopically negative surgical margins. Incomplete resection was defined as the presence of microscopically positive surgical margins (R1) and gross macroscopic residual tumor tissue (R2). OS was calculated from the date of diagnosis to death. PFS was calculated from the date of treatment initiation to the date of locoregional progression, distant metastasis, or death of any cause.

### Statistical analysis

Statistical analysis was performed using R language (R version 4.0.3). Kaplan–Meier survival curves were compared using the log-rank test. For descriptive statistics, continuous variables with normal distribution are presented as the mean ± standard deviation, whereas continuous variables with non-normal distribution are presented as median values (range). Categorical variables are described as counts and percentages. Statistical comparisons were made using paired t-test or unpaired, two-tailed t-test (as appropriate), with *p* < 0.05 deemed statistically significant. The survival benefit was measured using hazard ratios (HR) and its 95% confidence intervals (CI).

## Results

### Patient characteristics

After excluding 120 patients, 201 patients were included in the present study, of which 50 and 151 patients received TP and TC regimens, respectively. Table 1 summarizes the baseline characteristics of enrolled patients in both groups. The median age of the patients was 61 (IQR: 54–65) years, with a male to female ratio of 6.75. Most patients were aged < 65 years (71.1%). Considering the total population, most patients were smokers (n = 137, 68.2%) and consumed alcoholic beverages (n = 132, 65.7%). The tumor was mainly located in the lower esophagus (49.8%), and the proportion of patients with stage III disease was 73.1%. The patient characteristics in both groups were well balanced. There were no significant differences in age, sex, ECOG performance, tumor location, and clinical T and N stages. Comorbidities before the treatment such as diabetes, chronic obstructive pulmonary disease (COPD), hypertension, coronary heart disease (CHD) and hepatitis B mellitus were also investigated. The incidence was 4% (8/201) for diabetes, 1% (2/201) for COPD, 18.4% (37/201) for hypertension, 0.5% (1/201) for CHD and 3.5% (7/201) for hepatitis B. The comorbidities between the TC and TP groups were similar (Additional file 1: Table S1).

**Table 1:** Patient characteristics

Variables	Total (n = 201)	TP (n = 50)	TC (n = 151)	p
*Age, years, n(%)*				0.738
< 65	143 (71.1)	37 (74)	106 (70.2)	
≥ 65	58 (28.9)	13 (26)	45 (29.8)	
*Sex, n(%)*				0.339
Male	175 (87.1)	46 (92)	129 (85.4)	
Female	26 (12.9)	4 (8)	22 (14.6)	
*ECOG, n(%)*			0.778	
0	183 (91.0)	45 (90)	138 (91.4)	
1	18 ( 9.0)	5 (10)	13 (8.6)	
*Smoking, n(%)*			0.122	
Yes	137 (68.2)	39 (78)	98 (64.9)	
No	64 (31.8)	11 (22)	53 (35.1)	
*Drinking, n(%)*			0.36	
Yes	132 (65.7)	36 (72)	96 (63.6)	
No	69 (34.3)	14 (28)	55 (36.4)	
*Tumor location, n (%)*			0.284	
Uper	30 (14.9)	4 (8)	26 (17.2)	
Midle	71 (35.3)	19 (38)	52 (34.4)	
Lower	100 (49.8)	27 (54)	73 (48.3)	
*Clinical T stage, n (%)*			0.088	
T2	12 ( 6.0)	5 (10)	7 (4.6)	
T3	160 (79.6)	36 (72)	124 (82.1)	
T4a	17 ( 8.5)	3 (6)	14 (9.3)	
T4b	12 ( 6.0)	6 (12)	6 (4)	
*Clinical N stage, n (%)*			0.749	
N0	4 ( 2.0)	1 (2)	3 (2)	
N1	73 (36.3)	21 (42)	52 (34.4)	
N2	100 (49.8)	22 (44)	78 (51.7)	
N3	24 (11.9)	6 (12)	18 (11.9)	
*Stage, n (%)*			0.657	
II	8 ( 4.0)	3 (6)	5 (3.3)	
III	147 (73.1)	36 (72)	111 (73.5)	
IVA	46 (22.9)	11 (22)	35 (23.2)	

ECOG: Eastern Cooperative Oncology Group performance

### Toxicity of concurrent chemoradiotherapy

As shown in Table 2, 45 (86%) and 141 (93.4%) patients in the TP and TC groups, respectively, completed chemotherapy (*p* = 0.139). No significant differences in chemotherapy incompletion rates were noted between groups, mainly because patients could not tolerate chemoradiotherapy-related AEs. The TP group exhibited significantly higher hematologic (leukopenia, *p* = 0.006; neutropenia, *p* = 0.016; thrombocytopenia, *p* = 0.026) and non-hematologic AEs (nausea, *p* = 0.038; anorexia, *p* = 0.047; febrile neutropenia, *p* = 0.01) of all grades than the TC group (Additional file 1: Table S3). Hematologic and non-hematologic AEs of grade ≥ 3 were observed in 72 (35.8%) and 45 (22.4%) patients, respectively, in the intention-to-treat population (Table 2). Patients treated with TP (46%) presented a higher incidence of hematologic grade ≥ 3 AEs than those in the TC (32.5%) group, although the difference was not statistically significant (*p* = 0.118). The most notable difference was the incidence of leukopenia (46% *vs*. 28.5%, *p* = 0.054). The incidence of grade ≥ 3 non-hematologic AEs was significantly lower in the TC group than in the TP group (17.2 vs. 38%, *p* = 0.004), with particular differences in febrile neutropenia rates (10 vs. 1.3%, *p* = 0.011).

**Table 2:** Treatment compliance and major toxicities

Variables	Total (n = 201)	TP (n = 50)	TC(n = 151)	p
	n (%)	n (%)	n (%)	
Completed chemotherapy	184 (91.5)	43 (86)	141 (93.4)	0.139
Hematologic	72(35.8)	23 (46)	49 (32.5)	0.118
Leukopenia	68(33.8)	23 (46)	45(29.8)	0.054
Anemia	1( 0.5)	0(0)	1 (0.7)	1
Thrombocytopenia	4 ( 2.0)	2 (4)	2 (1.3)	0.259
Neutropenia	55 (27.4)	18 (36)	37(24.5)	0.162
Non-hematologic	45 (22.4)	19 (38)	26 (17.2)	0.004
Nauseau	17 ( 8.5)	7 (14)	10 (6.6)	0.139
Vomiting	9(4.5)	4(8)	5(3.3)	0.23
Esophagitis	8 ( 4.0)	4 (8)	4 (2.6)	0.108
Febrile neutropenia	7 ( 3.5)	5 (10)	2 (1.3)	0.011
Radiation pneumonitis	1 ( 0.5)	0 (0)	1 (0.7)	1
Hepatic dysfunction	1(0.5)	0(0)	1(0.7)	1

### Postoperative complications

Table 3 summarizes grade ≥ 3 postoperative complications according to the Clavien-Dindo classification. The overall incidence of complications was 26.4%, which was similar between the TP and TC group (20 vs. 28.5%, *p* = 0.32). The most frequent major postoperative complications were pleural effusion (12.4%), anastomotic fistula (7.5%) and pneumonia (5.5%). Overall, no significant between-group differences were observed in the incidence of postoperative complications.

**Table 3:** Postoperative complications

Variables	Total (n = 201)	TP(n = 50)	TC (n = 151)	p
	n (%)	n (%)	n (%)	
Postoperative complications	53 (26.4)	10 (20)	43 (28.5)	0.32
Pneumonia	11 (5.5)	1 (2)	10 (6.6)	0.298
Atelectasis	7 (3.5)	0 (0)	7 (4.6)	0.249
Pleural effusion	25 (12.4)	8 (16)	17 (11.3)	0.526
Chylothorax	2 (1.0)	0 (0)	2 (1.3)	1
Empyema	2 (1.0)	0 (0)	2 (1.3)	1
Respiratory failure	7 (3.5)	0 (0)	7 (4.6)	0.196
Heart failure	7 (3.5)	0 (0)	7 (4.6)	0.196
Anastomotic fistula	15 (7.5)	1 (2)	14 (9.3)	0.122

### Surgical outcomes

Among the 151 patients in the TC group, 146 (96%), 4 (2.6%), and 2 (1.4%) achieved R0, R1, and R2 resection, respectively. Forty-nine patients (98%) in the TP group achieved R0 resection, and 1 patient (2%) achieved R1 resection. Overall, 66 of 201 patients achieved pCR (32.8%). The pCR rate did not differ significantly between the TP and TC groups (38 vs. 31.1%, *p* = 0.469). No statistically significant intergroup differences were noted in terms of perineural invasion, lymphovascular invasion, resection margins, or pCR rates (all *p* > 0.05) (Table 4).

**Table 4:** Pathological findings

Variables	Total (n = 201)	TP (n = 50)	TC (n = 151)	p
*Perineural invasion, n (%)*				0.837
Negative	169 (84.1)	43 (86)	126 (83.4)	
Positive	32 (15.9)	7 (14)	25 (16.6)	
*Lymphovascular invasion, n (%)*				0.787
Negative	181 (90.0)	46 (92)	135 (89.4)	
Positive	20 (10.0)	4 (8)	16 (10.6)	
*Resection margins, n (%)*				1
R0	195 (96.5)	49 (98)	146 (96)	
R1	5 ( 2.5)	1 (2)	4 (2.6)	
R2	2 (1)	0 (0)	2 (1.4)	
*pCR, n (%)*				0.469
Yes	66 (32.8)	19 (38)	47 (31.1)	
No	135 (67.2)	31 (62)	104 (68.9)	
*Postop.T, n (%)*				0.82
T0	82 (40.8)	21 (42)	61 (40.4)	
T1-2	64 (31.8)	17 (34)	47 (31.1)	
T3-4	55 (27.4)	12 (24)	43 (28.5)	
*Postop.N, n (%)*				0.097
N0	140 (69.7)	40 (80)	100 (66.2)	
N +	61 (30.3)	10 (20)	51 (33.8)	

pCR: Pathologic complete remission

Of the 201 patients who received NCRT, 23.9% of patients did not show any downstaging, while 76.1% showed a decrease in at least one T stage. 88.1% patients showed a decrease in at least one N stage. The pathological response assessment was scored using the tumor regression grade (TRG) of the Becker criteria. As shown in the following table, 81 (40.3%), 46 (22.9%), 60 (29.9%), and 14 (7%) patients revealed no residual tumor (TRG 1a), < 10% residual tumor per tumor area (TRG 1b), 10%–50%residual tumor per tumor area (TRG 2), and > 50% residual tumor per tumor area (TRG 3) response in Additional file 1: Table S2, respectively. The differences of postoperative T/N stage downstaging and TRG score between the TP and TC groups were not statistically significant.

### Survival

**Fig. 1:**
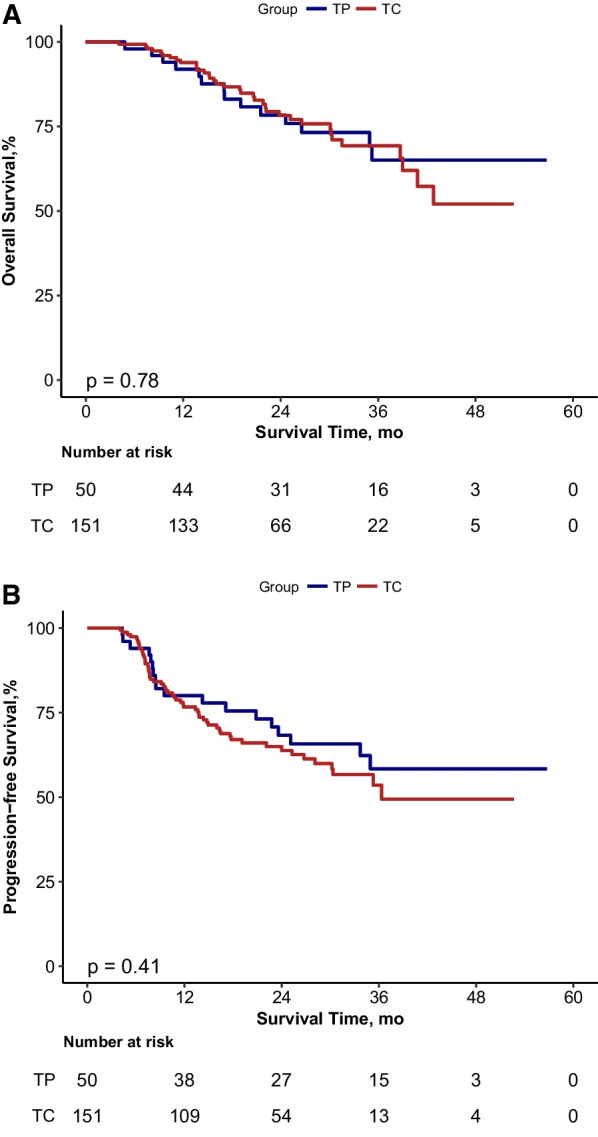
OS (**A**) and PFS (**B**) curve of TP and TC group

In total, 50 (24.9%) patients were dead at the end of the follow-up period. With a median follow-up of 26.9 months, the 3-year OS and 3-year PFS were 67.7 and 54.4%, respectively, for the entire population. There were no significant differences between the TP and TC groups considering OS (HR 1.093; 95%CI, 0.59–2.04; *p* = 0.78) and PFS (HR 1.252; 95%CI, 0.73–2.14; *p* = 0.41) (Fig. 1A, B). The 1-, 2-, and 3-year OS rates were 93.9, 78.3, and 69.4%, respectively, for patients in the TC groupand 92, 78.4, and 65.1%, respectively, for those in the TP group (Table 2). The 3-year PFS rates were 58.4 (95%CI, 44.7–76.3) and 53.5% (95%CI, 43.8–65.4) in the TP and TC groups, respectively.

We also investigated the outcome of the patients who received neoadjuvant treatment in the first place, but could not complete the treatment course. As shown in Table 2, total of 8.5% (17/201) patients did not complete chemotherapy. In these 17 patients, 4 (23.5%) of them were dead at the end of the follow-up period. Patients were separated into two groups according to completed chemotherapy or not for survival analysis. The 3-year OS rates were 68.3 (95%CI, 60.2–77.5) and 63.6% (95%CI, 38.2–100) in the complete chemotherapy and not complete groups, respectively. There were no significant differences between the two groups considering OS (HR 0.82; 95%CI, 0.29–2.28; *p* = 0.7) and PFS (HR 1.022; 95%CI, 0.47–2.23; *p* = 0.96).

## Discussion

To the best of our knowledge, this is the first study to compare TC and TP regimens in patients with LA-ESCC for NCRT. In the present retrospective study, we aimed to evaluate whether the TC regimen was superior to the TP regimen, which is a better chemotherapy regimen for NCRT in patients with LA-ESCC. Herein, we evaluated AEs and treatment-related effects of TC and TP regimens. The results suggest that either hematologic or non-hematologic AEs occurred less frequently in the TC group during NCRT. We noted no significant differences in postoperative complications. In addition, the incidence of pathological findings (including pCR, R0 resection rate and TRG grade) and survival outcomes were similar in both regimens. The TC regimen exhibits low toxicity and comparable efficacy.

The weekly TC regimen has afforded excellent efficacy in the CROSS study [10]. Since then, the carboplatin plan has been widely accepted and recommended by the National Comprehensive Cancer Network guidelines as the standard chemotherapy regimen for concurrent chemoradiotherapy, including neoadjuvant and definitive therapy. Regarding safety, patients in the TC group experienced mild AEs in the present study, with fewer grade 3 AEs and higher hematologic and gastrointestinal toxic effects than those in the TP group. However, toxic effects in the present study were more severe than those in the CROSS study, especially hematologic toxic effects (33.8% for over grade 3 leukopenia), which could be attributed to higher doses and fewer cycles of chemotherapy regimens. Herein, all patients who received the triweekly TC regimen completed two cycles of chemotherapy on an in-patient basis, which provided better management of the patient’s condition and AEs over a short period. Moreover, fewer chemotherapy cycles can result in larger financial benefits. However, patients who underwent weekly regimens mostly underwent outpatient chemotherapy. Conversely, a triweekly regimen is worthy of clinical consideration and application.

The nutritional status of patients with esophageal cancer tends to be poor, and malnutrition can lead to poor prognosis and death [18, 19]. Gastrointestinal AEs is a major factor in the aggravation of poor undernutrition condition in the treatment of patients with LA-ESCC. Thus, the management of adverse reactions in the alimentary tract is critical during chemoradiotherapy. Neoadjuvant therapy-related AEs can also have serious consequences, including failure to complete chemotherapy cycles, high risk of failure to progress to surgical resection, and poor OS [20–23]. In our study, the TP regmen shows higher rates of nausea (*p* = 0.038) and anorexia (*p* = 0.047), which result in a number of potential risk factors for malnutrition and poor prognosis. Therefore, we recommend the TC regimen as a superior alternative for NCRT.

Although the superiority of the TP regimen has been previously reported [11, 24, 25], a recent RCT has shown that TP does not afford better OS than TC regimens for definitive chemoradiation in patients with LA-ESCC [26]. However, higher rates of hematologic and gastrointestinal toxic effects were observed in the cisplatin group than in the carboplatin group. Similarly, another study has revealed that the TP regimen did not confer a survival advantage over other platinum-based therapies combined with paclitaxel in definitive chemoradiation [27]. Our findings support those reported in previous reports, and there was no significant difference in OS and PFS between the TC and TP regimens.

The limitations of the present study need to be addressed. First, this was a retrospective cohort study, with potential selection bias when compared with prospective randomized controlled studies. We attempted to match the two groups to eliminate the influence of bias and found that baseline characteristics were well balanced, implying that some bias may exist but remains insignificant. In addition, the follow-up durations were relatively short (mean follow-up duration, 26.9 months), and the median OS was not reached. Moreover, in our cohort, episodes of AEs were under-reported, given that this information was not prospectively gathered, and these mild events would not have warranted intervention or a change in treatment. A larger study comparing TC and TP regimens for NCRT in LA-ESCC is warranted.

## Conclusion

The findings of the present study indicate that the TC regimen is a safe and effective (equivalent) alternative to the TP regimen for NCRT in patients with LA-ESCC. Therefore, we recommend the TC regimen as a better option for NCRT. A future study comparing the TC and TP regimens for concurrent NCRT in LA-ESCC is warranted.

## Supplementary Information

**Additional file 1.** **sTable 1**. Comorbidities of the study population. **sTable 2**. Clinical pathological characteristics. **sTable 3**. All grades of major toxicities. **sTable 4**. Overall survival (OS) and disease-free survival (DFS) survival patients receiving TC or TP regimen.

## Data Availability

All data generated and analyzed during this study are included in this published article.

## Abbreviations

TP: Paclitaxel plus cisplatin
TC: Paclitaxel plus carboplatin
LA-ESCC: Locally advanced esophageal squamous cell carcinoma
NCRT: Neoadjuvant chemoradiotherapy
AEs: Adverse events
OS: Overall survival
PFS: Progression-free survival
HR: Hazard ratios
CI: Confidence intervals
ESCC: Esophageal squamous cell carcinoma
PF: Cisplatin plus 5-fluorouracil
RCTs: Randomized controlled trials
pCR: Pathological complete remission
AJCC: American Joint Committee on Cancer Staging System
ECOG: Eastern Cooperative Oncology Group
CTCAE: National Cancer Institute Common Terminology Criteria for Adverse Events
RT: Radiotherapy
GTV: Gross tumor volume
CT: Computed tomography
PET-CT: Positron emission tomography-computed tomography
CTV: Clinical target volume
IMRT: Intensity-modulated RT
RTOG: Radiation Therapy Oncology Group
