# Correction to: Safety and efficacy of paclitaxel plus carboplatin versus paclitaxel plus cisplatin in neoadjuvant chemoradiotherapy for patients with locally advanced esophageal carcinoma: a retrospective study

**DOI:** 10.1186/s13014-023-02245-0

**Published:** 2023-05-05

**Authors:** Li Jiang, Jie Zhu, Xue Chen, Yi Wang, Lei Wu, Gang Wan, Yongtao Han, Xuefeng Leng, Lin Peng, Qifeng Wang

**Affiliations:** 1 School of Medicine, Department of Radiation Oncology, Radiation Oncology Key Laboratory of Sichuan Province, Sichuan Cancer Center, School of Medicine, University of Electronic Science and Technology of China, Sichuan Cancer Hospital & Institute, University of Electronic Science and Technology of China, 55 South Renmin Ave, Fourth Section, Chengdu, Chengdu, Sichuan 610041 China; 2 Department of Radiation Oncology, Radiation Oncology Key Laboratory of Sichuan Province, Sichuan Cancer Center, School of Medicine, Sichuan Cancer Hospital & Institute, University of Electronic Science and Technology of China, 55 South Renmin Ave, Fourth Section, Chengdu, Sichuan 610041 China; 3 Department of Thoracic Surgery, Sichuan Cancer Center, School of Medicine, Sichuan Cancer Hospital & Institute, University of Electronic Science and Technology of China, Chengdu, China

**Radiation Oncology (2022) 17:218**

10.1186/s13014-022-02190-4

After publication of this article [1], the authors reported that in this article Li Jiang was incorrectly denoted as a corresponding author.

The original article [1] has been corrected.
