# Supplementary material for: Safety and efficacy of paclitaxel plus carboplatin versus paclitaxel plus cisplatin in neoadjuvant chemoradiotherapy for patients with locally advanced esophageal carcinoma: a retrospective study
Source: Radiat Oncol. 2022 Dec 30;17:218. doi: 10.1186/s13014-022-02190-4 (PMC9801619; doi:10.1186/s13014-022-02190-4)
Supplement: Supplementary file 1 — Additional file 1. sTable 1. Comorbidities of the study population. sTable 2. Clinical pathological characteristics. sTable 3. All grades of major toxicities. sTable 4. Overall survival (OS) and disease-free survival (DFS) survival patients receiving TC or TP regimen. [file 13014_2022_2190_MOESM1_ESM.docx]

**sTable 1. Comorbidities of the study population**

| Variables | Total (n = 201) | TP (n = 50) | TC (n = 151) | p |
| --- | --- | --- | --- | --- |
| Diabetes, n(%) | 8 ( 4.0) | 3 (6) | 5 (3.3) | 0.413 |
| COPD, n(%) | 2 ( 1.0) | 0 (0) | 2 (1.3) | 1 |
| Hypertension, n(%) | 37 (18.4) | 9 (18) | 28 (18.5) | 0.932 |
| CHD, n(%) | 1 ( 0.5) | 0 (0) | 1 (0.7) | 1 |
| Hepatitis B, n(%) | 7 ( 3.5) | 3 (6) | 4 (2.6) | 0.369 |

*COPD: Chronic obstructive pulmonary disease; CHD: Coronary heart disease.

**sTable 2. Clinical pathological characteristics.**

| Variables | Total (n = 201) | TP (n = 50) | TC (n = 151) | P |
| --- | --- | --- | --- | --- |
| TRG, n(%) |  |  |  | 0.876 |
| TRG 1a | 81 (40.3) | 21 (42) | 60 (39.7) |  |
| TRG 1b | 46 (22.9) | 13 (26) | 33 (21.9) |  |
| TRG 2 | 60 (29.9) | 13 (26) | 47 (31.1) |  |
| TRG 3 | 14 ( 7.0) | 3 (6) | 11 (7.3) |  |

*TRG: Tumor regression grade

**sTable 3. All grades of major toxicities.**

| Variables | Total (n = 201) | TP (n = 50) | TC (n = 151) | p |
| --- | --- | --- | --- | --- |
| Nauseau, n (%) | |  |  | 0.038 |
| 0 | 136 (67.7) | 26 (52) | 110 (72.8) |  |
| 1 | 33 (16.4) | 11 (22) | 22 (14.6) |  |
| 2 | 15 ( 7.5) | 6 (12) | 9 (6) |  |
| 3 | 17 ( 8.5) | 7 (14) | 10 (6.6) |  |
| Vomiting, n (%) | |  |  | 0.454 |
| 0 | 181 (90.0) | 44 (88) | 137 (90.7) |  |
| 1 | 3 ( 1.5) | 1 (2) | 2 (1.3) |  |
| 2 | 8 ( 4.0) | 1 (2) | 7 (4.6) |  |
| 3 | 9 ( 4.5) | 4 (8) | 5 (3.3) |  |
| anorexia, n (%) |  |  |  | 0.047 |
| 0 | 119 (59.2) | 23 (46) | 96 (63.6) |  |
| 1 | 34 (16.9) | 9 (18) | 25 (16.6) |  |
| 2 | 48 (23.9) | 18 (36) | 30 (19.9) |  |
| Malaise, n (%) |  |  |  | 0.588 |
| 0 | 198 (98.5) | 49 (98) | 149 (98.7) |  |
| 1 | 2 ( 1.0) | 1 (2) | 1 (0.7) |  |
| 2 | 1 ( 0.5) | 0 (0) | 1 (0.7) |  |
| Diarrhea, n (%) |  |  |  | 0.113 |
| 0 | 192 (95.5) | 46 (92) | 146 (96.7) |  |
| 1 | 8 ( 4.0) | 3 (6) | 5 (3.3) |  |
| 2 | 1 ( 0.5) | 1 (2) | 0 (0) |  |
| Febrile neutropenia, n (%) | |  |  | 0.01 |
| 0 | 194 (96.5) | 45 (90) | 149 (98.7) |  |
| 3 | 5 ( 2.5) | 4 (8) | 1 (0.7) |  |
| 4 | 2 ( 1.0) | 1 (2) | 1 (0.7) |  |
| Lung infection, n (%) | |  |  | 0.07 |
| 0 | 188 (93.5) | 44 (88) | 144 (95.4) |  |
| 1 | 6 ( 3.0) | 2 (4) | 4 (2.6) |  |
| 2 | 7 ( 3.5) | 4 (8) | 3 (2) |  |
| Radiation esophagitis, n (%) | |  |  | 0.372 |
| 0 | 73 (36.3) | 16 (32) | 57 (37.7) |  |
| 1 | 84 (41.8) | 22 (44) | 62 (41.1) |  |
| 2 | 36 (17.9) | 8 (16) | 28 (18.5) |  |
| 3 | 8 ( 4.0) | 4 (8) | 4 (2.6) |  |
| radiation pneumonitis, n (%) | |  |  | 0.55 |
| 0 | 176 (87.6) | 47 (94) | 129 (85.4) |  |
| 1 | 15 ( 7.5) | 2 (4) | 13 (8.6) |  |
| 2 | 9 ( 4.5) | 1 (2) | 8 (5.3) |  |
| 3 | 1 ( 0.5) | 0 (0) | 1 (0.7) |  |
| Hematologic, n (%) | |  |  | 0.004 |
| 0 | 18 ( 9.0) | 0 (0) | 18 (11.9) |  |
| 1 | 41 (20.4) | 11 (22) | 30 (19.9) |  |
| 2 | 70 (34.8) | 16 (32) | 54 (35.8) |  |
| 3 | 48 (23.9) | 11 (22) | 37 (24.5) |  |
| 4 | 24 (11.9) | 12 (24) | 12 (7.9) |  |
| Leukopenia, n (%) | |  |  | 0.006 |
| 0 | 28 (13.9) | 1 (2) | 27 (17.9) |  |
| 1 | 37 (18.4) | 11 (22) | 26 (17.2) |  |
| 2 | 68 (33.8) | 15 (30) | 53 (35.1) |  |
| 3 | 53 (26.4) | 15 (30) | 38 (25.2) |  |
| 4 | 15 ( 7.5) | 8 (16) | 7 (4.6) |  |
| Anemia, n (%) |  |  |  | 0.201 |
| 0 | 115 (57.2) | 26 (52) | 89 (58.9) |  |
| 1 | 73 (36.3) | 18 (36) | 55 (36.4) |  |
| 2 | 12 ( 6.0) | 6 (12) | 6 (4) |  |
| 3 | 1 ( 0.5) | 0 (0) | 1 (0.7) |  |
| Thrombocytopenia, n (%) | |  |  | 0.026 |
| 0 | 147 (73.1) | 30 (60) | 117 (77.5) |  |
| 1 | 33 (16.4) | 14 (28) | 19 (12.6) |  |
| 2 | 17 ( 8.5) | 4 (8) | 13 (8.6) |  |
| 3 | 3 ( 1.5) | 1 (2) | 2 (1.3) |  |
| 4 | 1 ( 0.5) | 1 (2) | 0 (0) |  |
| Neutropenia, n (%) | |  |  | 0.016 |
| 0 | 79 (39.3) | 14 (28) | 65 (43) |  |
| 1 | 32 (15.9) | 5 (10) | 27 (17.9) |  |
| 2 | 35 (17.4) | 13 (26) | 22 (14.6) |  |
| 3 | 31 (15.4) | 7 (14) | 24 (15.9) |  |
| 4 | 24 (11.9) | 11 (22) | 13 (8.6) |  |

**sTable 4. Overall survival (OS) and disease-free survival (DFS) survival patients receiving TC or TP regimen.**

| Regimen | | 1-year  (95% CI) (%) | 2-year  (95% CI) (%) | 3-year  (95% CI) (%) |
| --- | --- | --- | --- | --- |
| OS | TP | 92(84.7-99.8) | 78.4(67.3-91.3) | 65.1(51.1-83) |
|  | TC | 93.9(90.2-97.9) | 78.3(71-86.3) | 69.4(60.4-79.6) |
| DFS | TP | 80(69.6-91.9) | 68.3(56.1-83.1) | 58.4(44.7-76.3) |
|  | TC | 76.6(70.1-83.7) | 63.8(56-72.6) | 53.5(43.8-65.4) |
